# Extracellular vesicles isolated from *Trypanosoma cruzi* affect early parasite migration in the gut of *Rhodnius prolixus* but not in *Triatoma infestans*

**DOI:** 10.1590/0074-02760190217

**Published:** 2019-12-13

**Authors:** Larissa F Paranaiba, Alessandra A Guarneri, Ana C Torrecilhas, Maria N Melo, Rodrigo P Soares

**Affiliations:** 1 Universidade Federal de Minas Gerais, Departamento de Parasitologia, Belo Horizonte, MG, Brasil; 2 Fundação Oswaldo Cruz-Fiocruz, Instituto René Rachou, Belo Horizonte, MG, Brasil; 3 Universidade Federal de São Paulo, Departamento de Ciências Farmacêuticas, Diadema, SP, Brasil

**Keywords:** Trypanosoma cruzi, triatomines, extracellular vesicles, interaction

## Abstract

The protozoan *Trypanosoma cruzi* has the ability to spontaneously secrete extracellular vesicles (EVs). In this paper, *T. cruzi* EVs derived from epimastigote forms were evaluated during interaction with triatomine bugs *Rhodnius prolixus* and *Triatoma infestans*. *T. cruzi* EVs were purified and artificially offered to the insects prior to infection with epimastigote forms. No effect of EVs was detected in the parasite counts in the guts of both vectors after 49-50 days. On the other hand, pre-feeding with EVs delayed parasite migration to rectum only in the gut in *R. prolixus* after 21-22 days. Those data suggest a possible role of *T. cruzi* EVs during the earlier events of infection in the invertebrate host.

Chagas disease is caused by the protozoan *Trypanosoma cruzi* (Kinetoplastida: Trypanosomatidae). It is estimated that more than eight million people are infected and 25 million are at risk of acquiring the disease. More than 10 thousand people die per year due to complications of clinical manifestations of the disease. Chagas disease was originally found in the Americas, but recently, due to human migration, has expanded to non-endemic countries in North America, Europe and Asia. *T. cruzi* is transmitted by triatomines (Reduviidae: Triatominae) including *Triatoma infestans* and *Rhodnius prolixus*, the most important vectors in Latin America.^1^ ^,^ ^2^ ^,^ ^3^

To develop and establish infection within the hostile environments in the digestive tract of the vector and vertebrate hosts, *T. cruzi* developed a variety of strategies implicating a wide number of molecules.^4^ Those are important for attachment and internalisation including Tc-85,^5^ glycoinositolphospholipids^6^ and glycosylphosphatidylinositol(GPI)-mucins.^7^ Some of those molecules can be either shed or expressed in the surface of extracellular vesicles (EVs).

EVs are spontaneously released by any cells including prokaryotic and eukaryotic.^8^ Depending on the origin, size and function they can be classified as microvesicles, nanoparticles, apoptotic bodies and exosomes. In general, EVs are composed of a phospholipid bilayer containing lipids, proteins, glycoconjugates and nucleic acids.^9^ The role of EVs during interaction with vectors is poorly understood. However, it is already known that in another Trypanosomatid, *Leishmania major*, EVs are released by promastigotes in the midgut of the sand fly vector and further inoculated together with the parasite in the vertebrate host.^10^ Altogether, this inoculum will be important for cell attraction and parasite establishment in the vertebrate host. In this context, *T. cruzi* EVs have already demonstrated their pro-inflammatory activity during host innate and chronic immune responses.^11^ ^,^ ^12^ However, no study on the role during the interaction with triatomine vector was performed.

Here, we provide evidence that in the digestive tract of triatomine vetors, *T. cruzi* EVs were able to functionally affect early parasite migration in *R. prolixus* and had no effect on the number of metacyclics in both vector species.

Bug2149 cl10 *T. cruzi* strain (Bug), originally isolated from naturally infected *T. infestans* (Rio Grande do Sul, Brazil) was used.^13^ Epimastigote forms were cultured in liver-infusion tryptose (LIT) supplemented with 15% foetal bovine serum (FBS), 100 µg/mL streptomycin, 100 units/mL penicillin (27ºC and pH 7.2). *T. infestans* and *R. prolixus* used in this study were obtained from a laboratory colony derived from insects collected in Brazil and Honduras, respectively. Triatomines were reared at 25 ± 1ºC, 60 ± 10% relative humidity and natural illumination as previously reported.^14^ Fourth instar nymphs, starved for 30 days after ecdysis, were used in the assays.

Parasites were grown in LIT medium, washed in hanks’ balanced salt solution (HBSS), centrifuged (1000*g*/10min, 10ºC) and counted. For EVs release, *T. cruzi* in early log phase (1 x 10^5^ parasites/mL) were resuspended in LIT medium without FBS and incubated at 28ºC for 2 h. Parasites were fixed and cover slips were prepared for scanning electron microscopy (SEM) and transmission electron microscopy (TEM) as previously reported.^11^ ^,^ ^12^ After vesiculation, supernatants were collected, filtered (0.22 μm) and ultra-centrifuged (100,000*g*/2h, 4ºC). Nanoparticle tracking analysis (NTA) was performed to determine size, distribution and concentration of EVs as reported elsewhere.^12^ Acquisitions were measured in a Nanosight NS300 instrument (Malvern Instruments Ltd, Malvern, UK) equipped with a 405-nm laser and coupled to a charge-coupled device (CCD) camera (the laser emitting a 60-mW beam at 405-nm wavelength). Data were analysed using NTA software (version 2.3 build 0017). The detection threshold was set to 10. Blur, Min track Length and Min Expected Particle Size were set to auto. To perform the measurements, samples were diluted 1:100 in phosphate-buffered saline (PBS). Readings were taken in triplicates during 30 s at 20 frames per second (three times for each sample), at camera level set to 14 and manual monitoring of temperature (19ºC).

Citrated rabbit blood was obtained from Centro de Criação de Animais de Laboratório (CECAL), Fiocruz, RJ. Insects were artificially exposed to EVs and parasites in two consecutive moments: (i) first day, nymphs were artificially fed on citrated heat-inactivated (56ºC, 30 min) rabbit blood containing EVs. Each insect was allowed to ingest 20-30 μL of blood (approximately 6.4-9.6 x 10^4^ particles/µL); (ii) second day, the same nymphs were artificially fed to repletion on citrated heat-inactivated rabbit blood containing epimastigotes. Those parasites were obtained from LIT cultures, washed in PBS and resuspended in the rabbit blood at a final concentration of 1 x 10^7^ parasites/mL. Since each insect could ingest 20-30 mL of blood, the number of epimastigotes would range from 2-3 x 10^5^. Control group was fed on blood and blood + parasites with similar amounts of the treated group in the respective days. The midgut and rectum of a group of nymphs (n = 20) were individually dissected (Fig. 1) and homogenised in 30 μL of PBS (0.15 M NaCl at 0.01 M sodium phosphate, pH 7.4) at 21-22 days post infection (p.i.). Quantification of parasites was made in Neubauer chamber. At 28 days p.i., a new feeding with citrated heat-inactivated rabbit blood was offered for the remaining nymphs (n = 15). Immediately after feeding, the nymphs were transferred to 1.5 mL plastic tubes and the urine produced during diuresis was collected to evaluate the percentage of metacyclic forms. 10 µL of each sample were fixed in 10% methanol and stained with Giemsa®. The numbers of parasites were quantified by analysing the whole slide in optical microscopy. The same nymphs were dissected as described above at 49-50 days p.i. Sample size was calculated as reported elsewhere.^14^

**Fig. 1:**
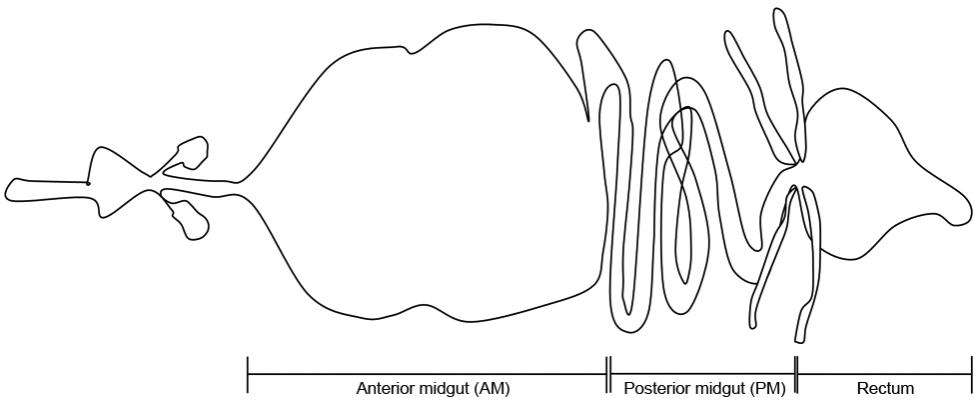
basic diagrammatic representation of a digestive tract from a triatomine bug.

To test whether or not the data follow a normal distribution, the test Kolmogorov-Smirnov was performed. Data showing a normal distribution were analysed by *t* test or analysis of variance (ANOVA). In the case of ANOVA, pairwise comparisons were performed by means of Tukey post hoc tests. Non-parametric data were analysed by Mann-Whitney test. P < 0.05 was considered significant.

*T. cruzi* epimastigotes were able to release EVs (Fig. 2A-B). Based on the data of the NTA, EVs exhibited an average size of 223.1 nm (D10 = 143.6; D50 = 245.5 and D90 = 264.7) (Fig. 2C) and a mean concentration between 6.84 x 10^7^ particles/mL (Fig. 2D). Released EVs were also subjected to TEM in order to confirm integrity and their sizes were within the range detected in NTA (Fig. 3A-D). For functional studies, those vesicles were mixed to rabbit blood and offered to the triatomines. After EVs exposure and infection, *T. cruzi* migration in different parts of the digestive tract was compared in two periods of infection (21-22 and 49-50 days p.i.). In *R. prolixus*, there was an increase in the number of parasites present in the midgut when compared to the control group at 21-22 days p.i. (Mann Whitney, p < 0.0001; Fig. 4A). In the rectum, however, the number of parasites was reduced in the nymphs treated with EVs (Mann Whitney, p < 0.0001; Fig. 4A). After 49-50 days p.i. the number of parasites in the midgut was reduced in both treatments, with no differences between the groups (Mann Whitney, n.s.; Fig. 4B). For *T. infestans*, no differences in the number of parasites between EVs-exposed nymphs and controls were observed (Mann Whitney, n.s.; Fig. 5A-B). At 28 days p.i. the number of metacyclis in the urine did not vary among controls and EV exposed insects for both triatomine species (*t* test, p > 0.05, Fig. 6A-B). All *T. infestans* nymphs released parasites in urine, but in numbers ~10 fold smaller than those found in *R. prolixus* ones.

**Fig. 2:**
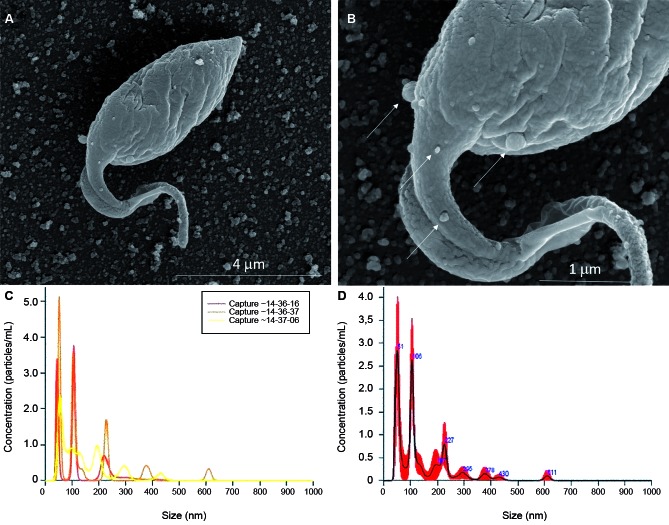
extracellular vesicles (EVs) of *Trypanosoma cruzi*. (A) Scanning electron microscopy (SEM) of *T. cruzi* membrane shedding (bars: 1-5 µm), Magnification 32,657 x and (B) Scanning electron microscopy (SEM) of *T. cruzi* membrane shedding (bars: 1-5 µm) Magnification 80,000 x. Nanoparticle tracking analysis (NTA) (C) and (D) of *T. cruzi* EVs.

**Fig. 3:**
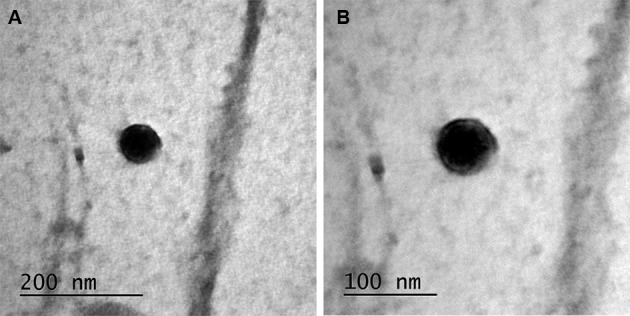
extracellular vesicles (EVs) of *Trypanosoma cruzi*. Transmission electron microscopy (TEM) of *T. cruzi* membrane shedding (A) (lower magnification, bar: 200 nm) and (B) (higher magnification, bar: 100 nm).

**Fig. 4:**
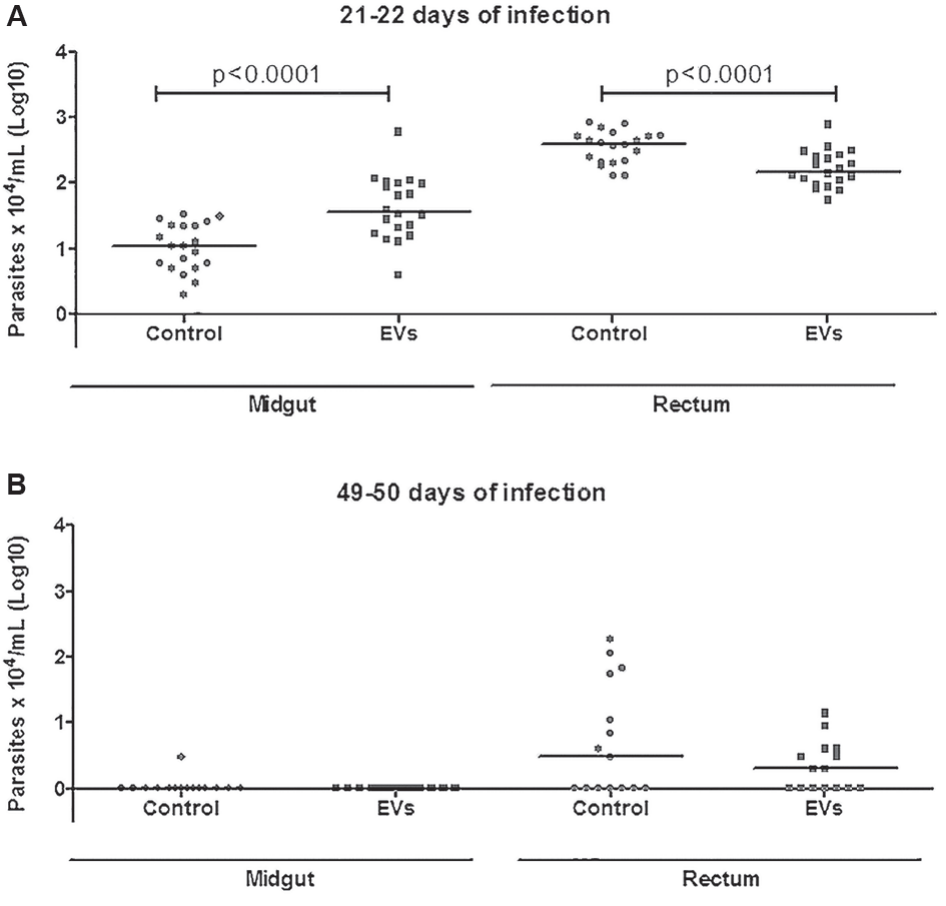
parasites found throughout the digestive tract of *Rhodnius prolixus*. Dissections at 21-22 (A) and 49-50 (B) days after the infective feeding. Each dot represents the quantification of parasites from one individual nymph and each horizontal bar corresponds to the median of the group evaluated.

**Fig. 5:**
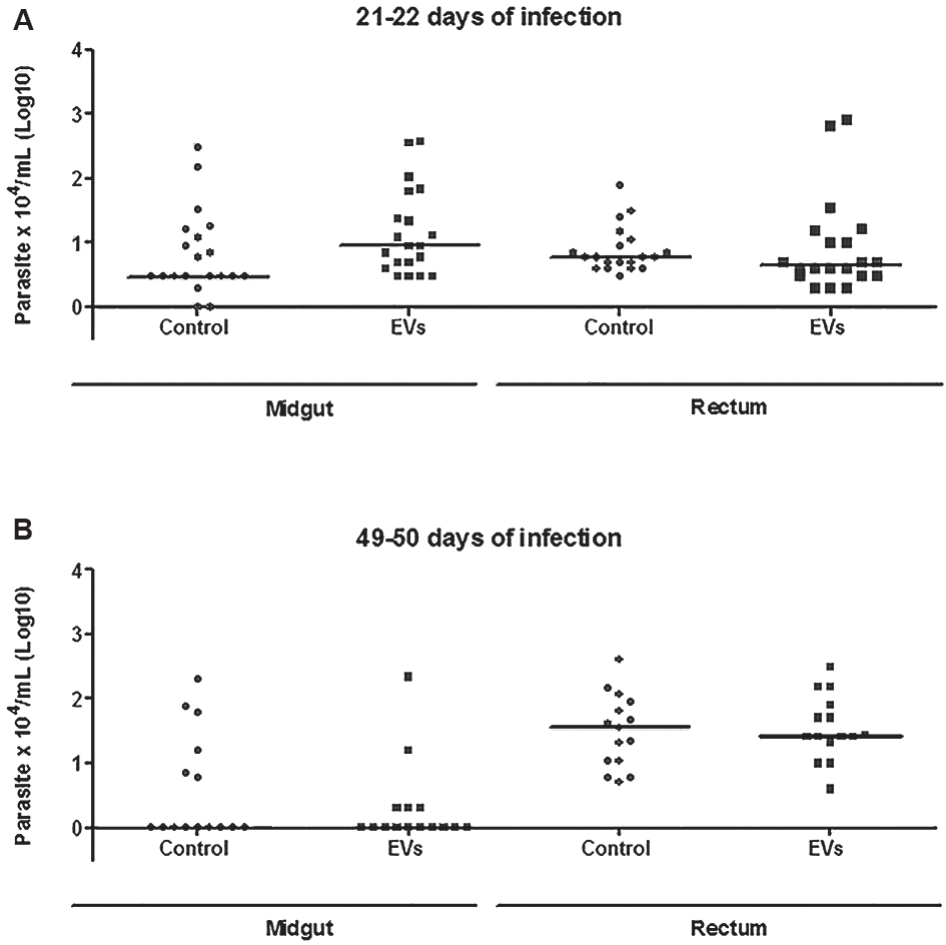
parasites found throughout the digestive tract of *Triatoma infestans*. Dissections at 21-22 (A) e 49-50 (B) days after the infective feeding. Each dot represents the quantification of parasites from one individual nymph and each horizontal bar corresponds to the median of the group evaluated.

**Fig. 6:**
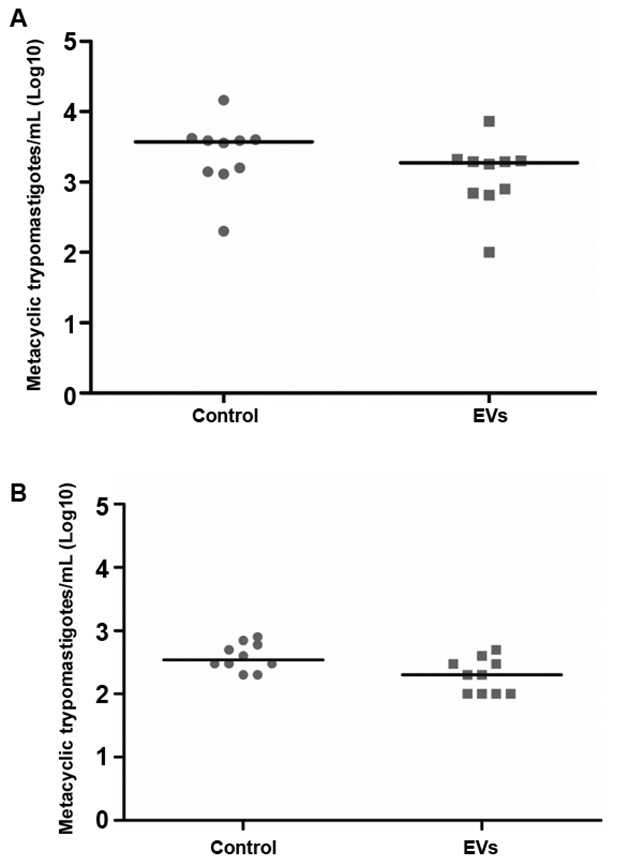
metacyclic trypomastigotes found in the urine of *Rhodnius prolixus* (A) and *Triatoma infestans* (B) at 28 days p.i. Each dot represents the quantification of parasites in the urine sample of one nymph and each horizontal bar corresponds to the mean of the group evaluated.

Parasites are known release exosome-like EVs that function as cell-to-cell effectors during the host-parasite interaction.^11^ ^,^ ^12^ ^,^ ^15^ ^,^ ^16^ ^,^ ^17^ One of the initial studies showed that challenge of BALB/c mice with *T. cruzi* EVs exacerbated parasite load, heart inflammation and mortality.^11^ Later, it was demonstrated that *T. cruzi* could modulated not only the innate but also the acquired immune events by activating TLR2, triggering cytokine production, MAPKs activation and invasion.^12^ ^,^ ^18^ It is important to mention that the concentration of vesicles used in those studies ranged from 1-10 µg/mL. Here, pre-feeding with EVs was approximately 5 µg/mL. Although it may be not physiological, this concentration is within a range known to functionally activate vertebrate cells. However, reports regarding the role of EVs with respect to their invertebrate hosts are unknown.

*R. prolixus* and *T. infestans* are the most used triatomine models due to due to existence of laboratory colonies. Both models were successfully used in our procedures and produced the expected infection pattern as previously reported.^14^ ^,^ ^15^ ^,^ ^16^ ^,^ ^17^ ^,^ ^18^ ^,^ ^19^ However, the ability of *T. infestans* to take a blood meal in the artificial system was much lower than that of *R. prolixus*. Pre-feeding with EVs delayed the early migration of *T. cruzi* parasites to the rectum (21-22 days p.i.) in *R. prolixus*. This effect was not observed after 49-50 days p.i., suggesting a transient effect of the EVs during the initial days of infection. However, this effect was not detected in *T. infestans* in both periods. The number of metacyclics in EV exposed and controls did not vary for both vectors reinforcing their role only in the initial events of infection. Interestingly, the number of metacyclic trypomastigotes recovered in the urine of *R. prolixus* was 10-fold higher than in *T. infestans*. This result was very surprising since Bug strain was originally isolated from *T. infestans* in Brazil, whereas the population of *R. prolixus* used in this study was from Honduras. *R. prolixus* belongs to the tribe Rhodniini, whereas *T. infestans* is from the Triatomini tribe.^20^ We thus believe that such differences may be attributed to the species rather than the amount of ingested blood. Although in our model, pre-feeding with EVs affected early parasite migration only in *R. prolixus*, their role in transmission was not assessed. Since the number of total parasites and metacyclics in later days of infection did not vary, it is not likely that EVs will affect transmission in triatomines. This is different from *L. major*, where EVs were important for parasite transmission during the sand fly bite.^10^ Despite the presence of EVs, we showed that both vectors were able to develop metacyclics. However, in nature those parasites are released in the feces and urine and are not inoculated as in *Leishmania*.

In vertebrate cells, it is already known that fusion of EVs is an important mechanism that promotes parasite internalisation.^11^ ^,^ ^18^ Our results suggest that *T. cruzi* EVs could be fusing to the epithelium of the midgut and somehow promoting early parasite retainment in *R. prolixus*. This effect was transient and did not affect the number of metacyclics in both vectors.
